# Open necrosectomy in acute pancreatitis–obsolete or still useful?

**DOI:** 10.1186/s13017-020-00300-9

**Published:** 2020-03-17

**Authors:** Henrik Leonard Husu, Jouni Antero Kuronen, Ari Kalevi Leppäniemi, Panu Juhani Mentula

**Affiliations:** 1 Department of Gastrointestinal Surgery, University of Helsinki and Helsinki University Hospital, P.O. Box 800, FI-00029 HUS Helsinki, Finland; 2 Medical Imaging Center, Helsinki University Hospital, P.O. Box 750, FI-00029 HUS Helsinki, Finland

**Keywords:** Necrosectomy, Open necrosectomy, Pancreatic necrosis, Walled-off necrosis, Infected pancreatic necrosis, Pancreatitis, Acute pancreatitis, Severe acute pancreatitis, Mortality, Organ failure

## Abstract

**Background:**

Multiple organ failure and early surgery are associated with high morbimortality after open necrosectomy. Data are mostly derived from historical cohorts with early necrosectomy bereft of step-up treatment algorithm implementation. Thus, mostly circumstantial evidence suggests a better clinical course following mini-invasive surgical and endoscopic necrosectomy. We studied the results of open necrosectomy in a contemporary cohort of patients with complicated pancreatic necrosis treated at a tertiary center.

**Methods:**

A retrospective cohort study from a university teaching hospital. Results of 109 consecutive patients treated with open necrosectomy during a 12-year period are reported.

**Results:**

The overall 90-day mortality rate was 22.9%. The 90-day mortality rate was 10.6% if necrosectomy could be delayed until 4 weeks from symptom onset and the necrosis had become walled off on preoperative imaging. The risk factors for 90-day mortality were age over 60 years (OR 19.4), pre-existing co-morbidities (OR 16.9), necrosectomy within 4 weeks (OR 6.5), multiple organ failure (OR 12.2), white blood cell count over 23 × 10^9^ (OR 21.4), and deterioration or prolonged organ failure as an indication for necrosectomy (OR 10.4). None or one of these risk factors was present in 52 patients (47.7% of all patients), and these patients had no mortality.

**Conclusion:**

Late open necrosectomy for walled-off necrosis has a low mortality risk. Open necrosectomy can be done without mortality in the absence of multiple risk factors for surgery.

## Background

Most patients with severe acute pancreatitis suffer from necrotizing pancreatitis, defined by the presence of (peri)pancreatic necrosis on computed tomography (CT) [1–3]. Infected pancreatic necrosis is associated with increased mortality risk and will mostly require invasive interventions [3–5]. A step-up management strategy should be implemented, whereby only patients with treatment failure after percutaneous or endoscopic drainage should be considered for debridement of necrotic tissue (necrosectomy) [6, 7]. All interventions should be postponed until 4 weeks into the disease whenever possible [6, 7].

Mortality rate after open necrosectomy varies between 8.8% and 22% in contemporary series [8–12]. Recent meta-analyses suggest similar short-term mortality in minimally invasive and open necrosectomy. However, open necrosectomy might be associated with increased adverse events and postoperative organ failure compared with minimally invasive necrosectomy, although the quality of evidence is low [13, 14]. Aspiration to alleviate surgical stress and minimize morbidity related to open necrosectomy has led to the development of less invasive debridement techniques. Minimally invasive necrosectomy is associated with an increased number of procedures relative to open necrosectomy [12]. Open necrosectomy provides thorough debridement in a single procedure, which may be advantageous especially in widespread necrosis. In patients who fail to improve or suffer complications after mini-invasive management of necrosis, open necrosectomy provides a bail-out treatment option. Based on recently published World Society of Emergency Surgery guidelines for the management of severe acute pancreatitis, open necrosectomy is still a valid treatment option for complicated pancreatic necrosis following a step-up treatment scheme [7]

The aim of this study was to determine mortality and morbidity risk factors following open necrosectomy in a contemporary cohort of patients.

## Methods

The study design was a retrospective analysis of all patients undergoing open surgical necrosectomy at Helsinki University Hospital (Meilahti Hospital) between 1 January 2006 and 31 December 2017. Meilahti Hospital, with a catchment population of 1.4 million, is a secondary and tertiary surgical referral center responsible for the majority of surgical necrosectomies in the capital area of Helsinki. Patients were identified from the database of surgical operations with ICD-10 diagnosis code K85.X and NOMESCO classification of surgical procedures code JLC50, which are used for pancreatic necrosectomy. Also, all patients with acute pancreatitis who were treated longer than 10 days or who had died within the treatment period were manually checked for potential necrosectomy. Data were collected from electronic hospital records, operation reports, laboratory sheets, and the radiological imaging database (Agfa IMPAX 6.6.1.5551). Severity of pancreatitis was classified according to the Revised Atlanta Classification of Pancreatitis Severity [2]. Radiological imaging prior to the first necrosectomy of all patients was re-evaluated by a radiologist (JK). The collected data included all interventions prior to the first necrosectomy from onset of disease, patient demographics, and other clinically relevant data. Patients were treated at the intensive care unit and/or surgical ward during their illness. Meilahti Hospital intensive care unit is administered by intensivists, but surgeons participate in the daily decision-making for pancreatitis patients. The first open surgical necrosectomy, i.e., debridement of pancreatic and/or peripancreatic necrotic tissue, serves as the index operation. Classification and assessment of risk factors are based on this index operation.

Positive bacterial culture from fine-needle aspiration or drainage of pancreatic necrosis or gas on CT scan in necrotic collection was considered a preoperative verification of infected pancreatic necrosis. Preoperative suspicion of infected pancreatic necrosis was based on clinicians’ judgment. Usually, clinicians’ suspicion arose in the event of elevated infectious laboratory parameters, new onset fever, or new onset organ failure at a later stage of the disease. If infected pancreatic necrosis was suspected, but could not be confirmed preoperatively, the microbiological samples taken from the pancreatic necrosis at the index operation determined whether or not the patient had infected pancreatic necrosis.

The method of choice at our center was mainly open necrosectomy. Although some endoscopic procedures and selected patients were treated with only percutaneous drainage of necrosis, these patients were not included in this study. Step-up methodology was implemented increasingly towards the latter part of the study period. Patients without preceding drainage were deteriorating, had a verified or suspected intra-abdominal emergency (e.g., bowel perforation or ischemia), were treated with open abdomen, did not have a safe percutaneous drainage route to the necrotic collection, or had widespread necrosis. The decision for surgical debridement was made by a multidisciplinary team with anaesthesiologists and presiding acute-care gastrointestinal surgeons.

A disconnected left pancreatic remnant on CT scan was defined according to Sandrasegaran et al. [15], meaning a large intrapancreatic non-enhancement combined with a viable segment of the distal body or tail of the pancreas. Data on all reoperations within 6 months of the index operation and endoscopic interventions up to 1 year were collected. If the patient survived to discharge, information on survival was retrieved from the Finnish Civil Registry. The STROBE statement was followed (www.strobe-statement.org).

### Surgical technique

Operations were mainly performed via upper transverse subcostal laparotomy because it provides good exposure to affected necrotic areas. However, midline laparotomy was used selectively in patients who have had laparostomy for treatment of abdominal compartment syndrome or in cases where bowel resection was anticipated. Pancreatic and peripancreatic necrosis was approached through the gastrocolic ligament. Necrosectomy was done using blunt manual dissection assisted with careful suction to avoid trauma to vital tissues. Microbiological samples from necrotic tissue were taken routinely during the operation. In cases where retrocolic or retromesenteric necrosis was present, these areas were approached lateral to the ascending or descending colon with or without mobilization of the hepatic flexure or splenic flexure, respectively. To decrease the risk of iatrogenic colonic injury, the mobilization of the colonic flexures was done only if it was deemed necessary for the debridement procedure. After debridement of all necrosis, lavage with normal saline was done. One to four Ch 24 silicone drains were left in place depending on the extent of the necrosis. A retroperitoneal route from flanks was used for the drains. Postoperative lavage was not used. In addition to necrosectomy, a disconnected left pancreatic remnant was resected, and a cholecystectomy was performed on patients with biliary pancreatitis during the index necrosectomy unless the patient was in poor condition. In addition, resection of necrotic or perforated bowel was done as needed. Open abdomen with vacuum-assisted closure after the operation was used only if closure of the abdomen was not possible.

### Statistical analysis

Statistical analysis was performed using SPSS 24.0 (IBM Corp., Armonk, NY, USA) and STATA 13.1 (StataCorp, College Station, TX, USA). Univariate odds ratios (ORs) with 95% confidence interval (CI) were calculated, and univariate logistic regression was used. Statistical testing of proportions was calculated using two-sided Fisher’s exact test and differences in continuous variables using Mann-Whitney *U* test. Receiver operating characteristic curve was used to define optimal cutoff points of continuous variables. Chi-square with linear-by-linear association test was utilized to calculate linear relationships. A multivariable logistic regression analysis based on univariate analysis of preoperatively available risk factors (*P* < 0.010) was performed. Multivariable logistic regression with stepwise forward method was used and goodness of fit of the regression model was tested with the Hosmer-Lemeshow test.

## Results

A systematic search of the database of surgical operations yielded 116 patients. A review of patient records revealed that three patients had procedures other than necrosectomy, three patients had the first necrosectomy done at another hospital, and one patient underwent laparoscopic necrosectomy; these seven patients were excluded from the study. Thus, 109 patients with primary open surgical necrosectomy for pancreatic necrosis were included in this study.

### Patient characteristics

Patient characteristics are summarized in Table 1. Of the 109 patients, 70 (64.2%) suffered from severe and 39 (35.8%) from moderately severe acute pancreatitis. At the time of the index necrosectomy, 44 patients (40.4%) were treated at the intensive care unit. The necrotic collection had become walled off in 91 patients (83.5%), whereas 18 patients (16.5%) suffered from acute necrotic collections according to preoperative CT. Twelve patients (11.0%) had single organ failure and 33 (30.3%) multiple organ failure within 24 h of necrosectomy. Preceding open abdomen treatment for abdominal compartment syndrome was necessary in 21 patients (19.3%). One of four acute care gastrointestinal surgeons performed the index operation on 94 patients (86.2%). The indication for necrosectomy was verified or suspected infected pancreatic necrosis in 82 patients (75.2%). Prolonged organ failure and/or deterioration of existing organ failures was the indication in 18 patients (16.5%). Six patients (5.5%) had necrosectomy due to prolonged pain and three patients (2.8%) due to bleeding, persistent gastric outlet obstruction symptoms, and suspicion of colonic necrosis. Ten patients (25.6%) between the years 2006 and 2011 and 30 patients (42.9%) between the years 2012 and 2017 were treated according to the step-up approach (*P* = 0.098). Within the study period, the annual proportion of patients undergoing step-up treatment increased significantly (*P* = 0.013).

**Table 1:** Patient characteristics at index necrosectomy

	(*n* = 109)
**Age at onset of symptoms, median (IQR), years**	52 (42–61)
**Time from onset of symptoms, median (IQR), days**	36 (22–59)
< 28 days from symptom onset	40 (36.7%)
**Length of stay at intensive care unit, median (IQR), days**	15 (1–24)
**Male sex**	96 (88.1%)
**Co-morbidities**	
Heart disease	22 (20.2%)
Pulmonary disease	10 (9.2%)
Mild renal insufficiency	4 (3.7%)
Diabetes	11 (10.1%)
Liver cirrhosis	2 (1.8%)
Chronic pancreatitis	3 (2.8%)
None of the above	66 (60.6%)
**Etiology**	
Alcohol	62 (56.9%)
Biliary	25 (22.9%)
Idiopathic	11 (10.1%)
Other*	11 (10.1%)
**Preoperative computed tomography**	
**Pancreatic necrosis**	
Not assessable	45 (41.3%)
< 30%	31 (28.4%)
30–50%	11 (10.1%)
> 50%	22 (20.2%)
**Distant pancreatic necrosis**^†^	98 (89.9%)
**Complex necrosis**^‡^	66 (60.6%)
**Disconnected left pancreatic remnant**	34 (31.2%)
**Previous organ failure**	
No organ failure	32 (29.4%)
< 48 h organ failure	7 (6.4%)
> 48 h organ failure	70 (64.2%)
**Previous interventions of necrosis**	
Fine-needle aspiration	31 (28.4%)
Percutaneous drainage	28 (25.7%)
Endoscopic drainage^§^	7 (6.4%)
Surgical drainage^||^	11 (10.1%)
Drainage duration, median (IQR), days^¶^	9 (6–14)
**CRP, median (IQR)**^#^	167 (89–290)
**WBC count, median (IQR)**^#^	12.6 (9.4–21.3)
**Intra-operative findings**	
Infected pancreatic necrosis	85 (78.0%)
Disconnected left pancreatic remnant	13 (11.9%)
**Resection of pancreas during index necrosectomy**	12 (11.0%)

*IQR* interquartile range, *CRP* C-reactive protein, *WBC* white blood cell

*Other: post-ERCP (6), postoperative (2), post-endoscopic (1), hypertriglyceridemia (1) and drug-induced (1)

^†^Local necrosis around pancreas, distant necrosis also in left/right paracolic gutter and/or retromesenteric area

^‡^Necrosis extending to both paracolic gutters or either of the paracolic gutters and the retromesenteric area

^§^Pseudocyst gastrostomy or transpapillary canalization

^||^Surgical canalization of necrosis in patients with existing abdomen treatment

^¶^Percutaneous or surgically placed drainage

^#^Within 24 h of index necrosectomy. CRP expressed as mg/L. WBC count expressed as 1 × 10^9^/L. Two CRP values and one WBC count were not taken 24 h prior to operation, and thus, the last available CRP value and WBC count prior to first necrosectomy, respectively, was used

In 98 patients (89.9%), the necrosis extended to either the paracolic gutter or the retromesenteric area. Sixty-six patients (60.6%) had necrosis in both paracolic gutters or one of the paracolic gutters and the retromesenteric area, which was defined as complex necrosis. A disconnected left pancreatic remnant was present in 40 patients (36.7%) on preoperative imaging or as an intraoperative finding at the index necrosectomy. Sixteen (40.0%) of these patients (11 simultaneously with the index necrosectomy) were treated with a pancreatic resection within 6 months. Pancreatico- or fistulojejunostomy was performed on 4 (10.0%) of 40 patients with a disconnected left pancreatic remnant. Endoscopic stenting of the pancreatic duct following open necrosectomy was performed on 15 patients (37.5%) with a disconnected left pancreatic remnant. Pancreatic fistula following necrosectomy was observed in 43 patients (39.4%), significantly more often in patients with a disconnected left pancreatic remnant on preoperative CT scan (*P* = 0.007). Simultaneously with the first necrosectomy, 20 cholecystectomies, 7 bowel resections, 7 splenectomies, and 12 pancreatic resections were performed. Synchronous cholecystectomy with the index operation was performed on 15 patients (60%) with biliary pancreatitis.

### Risk of infected pancreatic necrosis

Pancreatic necrosis was infected in 85 patients (78.0%). The probability for infected pancreatic necrosis was higher when necrosectomy was done after 4 weeks from symptom onset than when necrosectomy was performed within the first 4 weeks (*P* = 0.017). All patients with open abdomen before the first necrosectomy developed infected pancreatic necrosis, whereas 64 patients (72.7%) without antecedent open abdomen treatment developed infected pancreatic necrosis (*P* = 0.006).

### Postoperative organ failure

A new onset organ failure within 1 week following the index operation occurred in 38 patients (34.9%). In 15 patients (13.8%), the organ failure was transient, resolving within 48 h, whereas 23 patients (21.1%) had a new persistent organ failure that did not resolve within 48 h. No association emerged between new onset organ failure and 90-day mortality (Table 2). A total of seven organ failures recorded before the first necrosectomy (in five different patients) resolved within 48 h of the first necrosectomy.

**Table 2:** Postoperative predictors of mortality

Risk factor	Survivors (*n* = 84)*	Non-survivors (*n* = 25)*	OR (95% CI)	*P*
**New onset organ failure**	26 (31.0%)	12 (48.0%)	2.059 (0.828–5.120)	0.152
Persistent organ failure	15 (17.9%)	8 (32.0%)	2.165 (0.789–5.937)	0.163
**Multiple necrosectomies**	18 (21.4%)	9 (36.0%)	2.063 (0.783–5.434)	0.186
**Other reoperations**	27 (32.1%)	15 (60.0%)	3.167 (1.260–7.961)	**0.018**
Postoperative bleeding^†^	3 (3.6%)	8 (32.0%)	12.706 (3.052–52.893)	**< 0.001**
Enteric fistula or intestinal ischaemia^‡^	10 (11.9%)	10 (40.0%)	4.933 (1.748–13.922)	**< 0.003**
**Postoperative open abdomen**	12 (14.3%)	14 (56.0%)	7.636 (2.813–20.728)	**< 0.001**

*Percentage in parentheses is % of survivors/non-survivors

^†^Reoperation due to postoperative bleeding

^‡^Indication for reoperation: verified or suspected enteric fistula or intestinal ischemia

### Renecrosectomies and other reoperations

Indications and reoperations are presented in Additional file 1. Overall, 52 patients (47.7%) had a reoperation, and 27 patients (24.8%) had a renecrosectomy within 6 months of the index operation. Twenty-seven (67.5%) of 40 patients needed at least one additional operation (renecrosectomy or other) if the first necrosectomy was within 28 days of symptom debut, whereas 25 (36.2%) of 69 patients required an additional operation if necrosectomy could be postponed for more than 28 days (*P* = 0.003). Complex necrosis increased the risk of any reoperation, as 38 patients (57.6%) with complex necrosis underwent a reoperation compared with 14 patients (32.6%) without complex necrosis (*P* = 0.012).

Median period from symptom onset to the first necrosectomy was 40 days (interquartile range, IQR, 25–67) if patients underwent one, and 25 days (IQR 19-36) if patients underwent more than one necrosectomy (*P* = 0.006). First necrosectomy within 28 days (OR 3.52, 95% CI 1.43–8.67, *P* = 0.010), open abdomen at the time of index necrosectomy (OR 11.47, 95% CI 3.21–40.96, *P* < 0.001), postoperative open abdomen treatment (OR 8.07, 95% CI 3.00–21.71, *P* < 0.001), preceding multiple organ failure (OR 7.01, 95% CI 2.70–18.19, *P* < 0.001), and severe acute pancreatitis at the time of the first necrosectomy (OR 3.12, 95% CI 1.07–9.05, *P* = 0.038) were associated with the need for repeat necrosectomies.

Other reoperations were performed on 42 patients (38.5%). The need for reoperations was associated with the need for renecrosectomies (*P* = 0.006). Index necrosectomy due to acute necrotic collections increased the risk for a reoperation due to bleeding compared with necrosectomy for walled-off necrosis (*P* = 0.002). Nine patients (8.3%) needed a second operation for the treatment of pancreatic fistula.

### Ninety-day mortality after necrosectomy

**Fig. 1:**
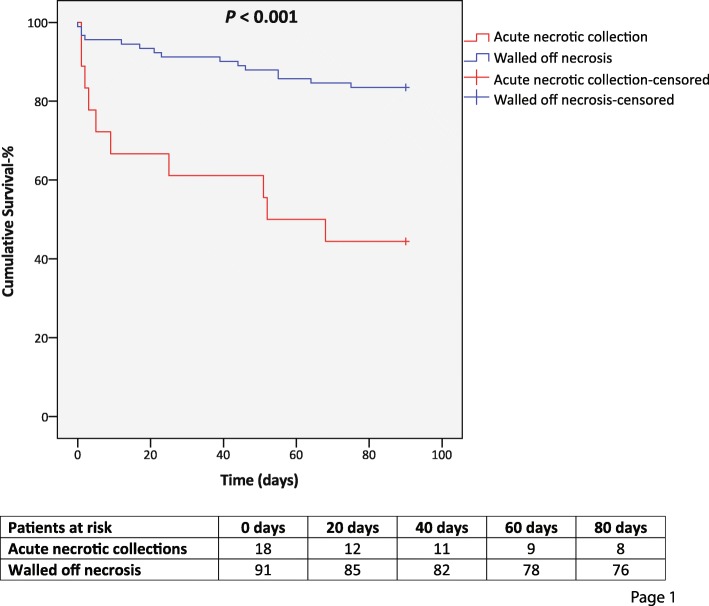
Kaplan-Meier 90-day survival table, walled-off necrosis vs. acute necrotic collections

Overall, 25 patients (22.9%) died within 90 days of the first necrosectomy (Fig. 1). The median age at disease onset was 50 years (IQR 40–59) in survivors and 61 years (52–67) in non-survivors (*P* = 0.002). Age over 60 years, necrosectomy within 28 days, acute necrotic collections, multiple organ failure, preoperative white blood cell (WBC) count over 23 × 10^9^, deterioration or prolonged organ failure as an indication for necrosectomy, and leaving pancreatic necrosis during necrosectomy were associated with increased mortality in univariate analysis (Additional file 2). When necrosectomy for walled-off necrosis could be delayed until 4 weeks after onset of symptoms, 7 (10.6%) of 66 patients died, whereas 8 (32.0%) of 25 patients died if necrosectomy was carried out earlier (*P* = 0.024). Postoperative risk factors for death are summarized in Table 2.

Independent risk factors for 90-day mortality were age over 60 years, any co-morbidity, deterioration or prolonged organ failure as the indication for necrosectomy, necrosectomy within 28 days of symptom onset, multiple organ failure at the time of necrosectomy, and WBC count over 23 × 10^9^ (Table 3). Based on the result of multivariate analysis, the effects of different risk factor combinations on 90-day mortality were calculated. As shown in Table 4, any combination of these six risk factors increased the mortality rate to at least 50%, whereas in 52 patients who had no more than one risk factor, the mortality rate was 0%.

**Table 3:** Ninety-day survival, forward conditional multivariate analysis

Risk factor	OR (95% CI)	*P*
Age > 60 years	19.355 (2.466–151.593)	**0.005**
Any co-morbidity*	16.869 (1.981–143.633)	**0.010**
Indication of necrosectomy: deterioration/prolonged organ failure	10.421 (1.572–69.080)	**0.015**
Necrosectomy < 28 days from symptom onset	6.480 (1.280–32.812)	**0.024**
Multiple organ failure^†^	12.159 (1.155–127.981)	**0.038**
Preoperative WBC count ≥ 23.0^‡^	21.442 (3.162–145.392)	**0.002**

Cut-off value for entry into multivariate analysis was *P* < 0.010 in Additional file 2

*CI* confidence interval, *WBC* white blood cell

*Any of the comorbidities presented in univariate analysis of all patients (Additional file 2)

^†^At least two of the following organ failures within 24 h of first necrosectomy: Cardiovascular, respiratory or renal

^‡^WBC count expressed as 1 × 10^9^/L. One WBC count was not taken 24 h prior to operation, and thus, the last available WBC count prior to first necrosectomy was used

**Table 4:** Risk factor combinations and mortality

	Age > 60 years	Co-morbidity*	Indication^†^	Necrosectomy < 28 days^‡^	Multiple organ failure	WBC count ≥ 23.0^§^	No risk factors^||^
**Age > 60 years**	**41.9%**, 31						
Co-morbidity*	**50.0%**, 18	**32.6%**, 43					
**Indication**^†^	**85.7%**, 7	**50.0%**, 6	**66.7%**, 18				
**Necrosectomy < 28 days**^‡^	**61.5%**, 13	**60.0%**, 15	**88.9%**, 9	**42.5%**, 40			
**Multiple organ failure**	**83.3%**, 6	**75.0%**, 8	**84.6%**, 13	**52.4%**, 21	**48.5%**, 33		
**WBC count ≥ 23.0**^§^	**100.0%**, 6	**80.0%**, 10	**88.9%**, 9	**76.9%**, 13	**68.8%**, 16	**62.5%**, 24	
**No risk factors**^||^	**0.0%**, 6	**0.0%**, 11	**0.0%**, 2	**0.0%**, 5	**0.0%**, 2	**0.0%**, 2	**0.0%**, 24

Bolded values represent mortality percentage at 90-day follow up after first necrosectomy in patients with the specific risk factor combination in question. Non-bolded values are the absolute number of patients with the specific risk combination in question

*WBC* white blood cell

*Any of the gathered co-morbidities in univariate analysis (Additional file 2)

^†^Indication of necrosectomy: deterioration/prolonged organ failure

^‡^From symptom onset

^§^WBC count expressed as 1 × 10^9^/L. Within 24 h of first necrosectomy. One WBC count was not taken 24 h prior to operation, and thus, the last available WBC count prior to first necrosectomy was used

^||^None of the risk factors presented in multivariate analysis (Table 3)

## Discussion

This study shows that mortality after open necrosectomy depends on patients’ preoperative risk factors. Preoperative risk factors for open necrosectomy were age over 60 years, pre-existing co-morbidities, necrosectomy within 4 weeks, multiple organ failure, white blood cell (WBC) count over 23 × 10^9^, and deterioration or prolonged organ failure as an indication for necrosectomy. The absence of specified risk factor combinations facilitates open necrosectomy without mortality. On the other hand, mortality risk is high when these risk factor combinations are present. We showed that the indication for open necrosectomy is associated with mortality, and that mortality risk increased if patients were deteriorating or when patients did not have clinical improvement of organ failures preceding open necrosectomy. We report that the mortality rate after open necrosectomy for walled-off necrosis is around 10% if the procedure can be postponed for the first 28 days.

Our study shows that patients without multiple risk factors for surgery have excellent results after open necrosectomy, as there was no mortality in this subgroup of patients. Consequently, diminishing these preoperative risk factors will improve outcome. From a practical standpoint, the only risk factors that could be manipulated are timing and indication for surgery. In line with treatment guidelines, open necrosectomy should be postponed as long as possible—for at least the first four weeks—if the patient can tolerate the complicated pancreatitis [6, 7]. If multiple organ failure prevails for weeks or the patient deteriorates despite maximal supportive care, mortality after open surgery is high when the patient has co-existing risk factors for open necrosectomy. Whether a non-operative approach or minimally invasive techniques would result in better outcomes in these patients warrants investigation since sparse data are available on their management.

Based on the current literature and our own experience, we summarize recommendations for the use of open necrosectomy in a modern-day setting (Table 5). Open necrosectomy serves as a salvage technique for complications following other interventions or the (peri)pancreatic necrosis itself and is a treatment option after treatment failure following a minimally invasive step-up algorithm. An ongoing open abdomen treatment, the need for simultaneous other resections (e.g., resection of a disconnected left pancreatic remnant), and available local expertise are other reasons for use of the open necrosectomy technique.

**Table 5:** Situations in which open necrosectomy is a valid option for treatment of infected pancreatic necrosis

Treatment failure or complication (e.g., persistent bleeding after attempted endovascular treatment) after step-up management procedure	
Bowel ischemia or perforation (suspected/verified) due to necrosis	
Ongoing open abdomen with simultaneous indication for necrosectomy	
Disconnected left pancreatic remnant fueling the disease	
Insufficient experience or equipment for mini-invasive necrosectomy	
Biliary pancreatitis with simultaneous need for cholecystectomy	
Anatomically widespread necrosis	

The overall mortality rate reported in our study is in line with recent reported cohorts of patients undergoing open necrosectomy [8–11]. Consistent with previous studies, our results indicate that age, multiple organ failure, and timing of intervention dramatically affect mortality [10, 11, 16, 17]. An association between markedly increased WBC count and death has earlier been shown in studies of acute pancreatitis and severe acute pancreatitis [18–21].

There was significant morbidity in terms of a need for additional operations after the first open necrosectomy. However, all patients regardless of the complexity of necrosis, timing of surgery, or indication for surgery were included in this study. Around two-thirds of our patients had complex necrosis (affecting both paracolic gutters or the retromesenteric area and one of the paracolic gutters), which was associated with the need for re-interventions. We treated 14 patients with an ongoing open abdomen at the time of the index operation, and one-third of all patients had multiple organ failure within 24 h of the first necrosectomy; both of these factors were significantly associated with later morbidity in this study. Hence, it seems that the severity of the disease and the extent of necrosis probably increased the need for re-interventions. At least in part due to the historical nature of this series, the first necrosectomy was carried out within 28 days of symptom debut in more than one-third of patients. As shown by our research, early necrosectomy was significantly associated with the need for additional interventions.

The rate of postoperative pancreatic fistulas was comparable to previous studies on open and mini-invasive surgical necrosectomy and clearly higher than after endoscopic or transgastric open surgical necrosectomy [8, 22, 23]. On the other hand, our study showed that only a minority of patients with postoperative pancreatic fistulas needed an additional operation, and most patients could be treated by endoscopic stenting. In the context of open surgery, open transgastric necrosectomy seems to be a beneficial strategy to tackle the high incidence of pancreaticocutaneous fistulation after open surgery and might reduce the need for resection of the tail of the pancreas after a disconnected left pancreatic remnant [22].

One-quarter of all patients required a median of one additional necrosectomy. Conversely, 75% of patients had no need for repeat necrosectomy, which is in accord with previous studies [8, 9]. Mini-invasive techniques, such as video-assisted retroperitoneal debridement and endoscopic necrosectomy, are associated with the need for repeat debridement procedures due to insufficient initial debridement [24, 25] Open necrosectomy probably facilitates the most complete debridement of pancreatic necrosis in a single procedure. Interestingly, our results indicate that failure to fully debride necrosis is a significant risk factor for death, and hence, a minimally invasive repeat debridement strategy might be disadvantageous in patients with widespread necrosis.

During the study period percutaneous drainage was not extensively used. We implemented step-up management to postpone surgical intervention when the necrotic collection had not matured and if there was suspicion of infected pancreatic necrosis with a diagnostic and therapeutic intention. In case of clinical deterioration of the patient or when an abdominal catastrophe was suspected or verified, primary open necrosectomy was employed. The publication of the PANTER trial of step-up mini-invasive necrosectomy in 2010 is seemingly reflected in our results as an increased use of percutaneous drainage towards the latter years of our study period [8]. However, most patients in our study suffered from complex pancreatic necrosis, which has been associated with drainage treatment failure in previous studies [26].

This study has several limitations. The retrospective setting, in which the indications and timing for surgery were not controlled, may introduce a bias to the range of patients treated. However, in contrast to several other reports regarding open, mini-invasive, and endoscopic necrosectomies, we included the whole spectrum of patients treated at our institution, including the most severe cases with preceding open abdomen treatment for abdominal compartment syndrome. Most patients had a widespread necrosis affecting beyond the area around the pancreas, and a disconnected left pancreatic remnant was radiologically evaluated to be present in about one-third of cases. Both of these factors reflect the severity of disease treated in this cohort. Also, the annual number of necrosectomies in this cohort was fairly low, averaging 9 cases a year over the 12-year study period, which would computationally account for less than 5% of patients with acute pancreatitis [1]. It therefore seems unlikely that the threshold for surgery was too low at our center. Due to the inherent nature of the retrospective setting of this study, we cannot rule out unknown confounding factors, e.g., utilization of intensive care unit treatment. However, we are not aware of any significant other management changes, such as availability of intensive care unit treatment, during the study period. Another limitation is that organ-specific severity was not evaluated systematically from the cohort, which would have shed more light on the severity of disease at the time of surgery, albeit around 40% of patients had single or multiple organ failure at the time of surgery. Furthermore, although a clinical follow-up visit for the patients was arranged, there was no specific or pre-adjusted schema for these outpatient controls, and thus, information regarding, for example, hernia occurrence, use of pancreatic enzymes, rate of new onset diabetes, and measures of quality of life is lacking.

## Conclusion

Open necrosectomy is a viable treatment in selected patients with complicated pancreatic necrosis. Patients with walled-off necrosis who are treated with open necrosectomy after 28 days from disease onset have around 10% mortality. Mortality is higher than 50% when multiple risk factors for open necrosectomy are present. Without these multiple risk factors, open necrosectomy can be done without mortality.

## Supplementary information

**Additional file 1:.** Additional Table 1. Reoperations Within 6 Months and Endoscopic/Other Procedures Within One Year After First Necrosectomy.

**Additional file 2:.** Additional Table 2. Univariate Analysis of 90-day Mortality.

## Data Availability

We have not filed for permission to publish the study material.

## Abbreviations

CI: Confidence interval
CT: Computed tomography
IQR: Interquartile range
OR: Odds ratio
WBC: White blood cell

## References

[1] Lankisch Paul Georg, Apte Minoti, Banks Peter A (2015). Acute pancreatitis. The Lancet.

[2] Banks PA, Bollen TL, Dervenis C, Gooszen HG, Johnson CD, Sarr MG (2013). Classification of acute pancreatitis--2012: revision of the Atlanta classification and definitions by international consensus. Gut.

[3] Banks PA, Freeman ML (2006). Practice guidelines in acute pancreatitis. Am J Gastroenterol.

[4] Petrov Maxim S., Shanbhag Satyanarayan, Chakraborty Mandira, Phillips Anthony R.J., Windsor John A. (2010). Organ Failure and Infection of Pancreatic Necrosis as Determinants of Mortality in Patients With Acute Pancreatitis. Gastroenterology.

[5] van Santvoort HC, Bakker OJ, Bollen TL, Besselink MG, Ahmed Ali U, Schrijver AM (2011). A conservative and minimally invasive approach to necrotizing pancreatitis improves outcome. Gastroenterology.

[6] Working Group IAP/APA Acute Pancreatitis Guidelines. IAP/APA evidence-based guidelines for the management of acute pancreatitis. Pancreatology. 2013;13, 1(4 Suppl 2). 10.1016/j.pan.2013.07.063. 10.1016/j.pan.2013.07.063 24054878

[7] Leppäniemi A, Tolonen M, Tarasconi A, Segovia-Lohse H, Gamberini E, Kirkpatrick AW (2019). 2019 WSES guidelines for the management of severe acute pancreatitis. World J Emerg Surg.

[8] van Santvoort HC, Besselink MG, Bakker OJ, Hofker HS, Boermeester MA, Dejong CH (2010). A step-up approach or open necrosectomy for necrotizing pancreatitis. N Engl J Med.

[9] Babu BI, Sheen AJ, Lee SH, O'Shea S, Eddleston JM, Siriwardena AK (2010). Open pancreatic necrosectomy in the multidisciplinary management of postinflammatory necrosis. Ann Surg.

[10] Madenci AL, Michailidou M, Chiou G, Thabet A, Fernandez-del Castillo C, Fagenholz PJ (2014). A contemporary series of patients undergoing open debridement for necrotizing pancreatitis. Am J Surg.

[11] Gomatos IP, Halloran CM, Ghaneh P, Raraty MG, Polydoros F, Evans JC (2016). Outcomes from minimal access retroperitoneal and open pancreatic necrosectomy in 394 patients with necrotizing pancreatitis. Ann Surg.

[12] van Brunschot S, Hollemans RA, Bakker OJ, Besselink MG, Baron TH, Beger HG (2018). Minimally invasive and endoscopic versus open necrosectomy for necrotising pancreatitis: a pooled analysis of individual data for 1980 patients. Gut.

[13] Cirocchi R, Trastulli S, Desiderio J, Boselli C, Parisi A, Noya G (2013). Minimally invasive necrosectomy versus conventional surgery in the treatment of infected pancreatic necrosis: a systematic review and a meta-analysis of comparative studies. Surg Laparosc Endosc Percutan Tech.

[14] Gurusamy KS, Belgaumkar AP, Haswell A, Pereira SP, Davidson BR (2016). Interventions for necrotising pancreatitis. Cochrane Database Syst Rev.

[15] Sandrasegaran Kumaresan, Tann Mark, Jennings S. Gregory, Maglinte Dean D., Peter Sanjit D., Sherman Stuart, Howard Thomas J. (2007). Disconnection of the Pancreatic Duct: An Important But Overlooked Complication of Severe Acute Pancreatitis. RadioGraphics.

[16] Mofidi R, Lee AC, Madhavan KK, Garden OJ, Parks RW (2007). Prognostic factors in patients undergoing surgery for severe necrotizing pancreatitis. World J Surg.

[17] Raraty MG, Halloran CM, Dodd S, Ghaneh P, Connor S, Evans J (2010). Minimal access retroperitoneal pancreatic necrosectomy: improvement in morbidity and mortality with a less invasive approach. Ann Surg.

[18] De Campos T, Cerqueira C, Kuryura L, Parreira JG, Solda S, Perlingeiro JA (2008). Morbimortality indicators in severe acute pancreatitis. JOP.

[19] Kaya E, Dervisoglu A, Polat C (2007). Evaluation of diagnostic findings and scoring systems in outcome prediction in acute pancreatitis. World J Gastroenterol.

[20] Al Mofleh IA (2008). Severe acute pancreatitis: pathogenetic aspects and prognostic factors. World J Gastroenterol.

[21] Zhao JG, Liao Q, Zhao YP, Hu Y (2014). Mortality indicators and risk factors for intra-abdominal hypertension in severe acute pancreatitis. Int Surg.

[22] Driedger Michael, Zyromski Nicholas J., Visser Brendan C., Jester Andrea, Sutherland Francis R., Nakeeb Atilla, Dixon Elijah, Dua Monica M., House Michael G., Worhunsky David J., Munene Gitonga, Ball Chad G. (2020). Surgical Transgastric Necrosectomy for Necrotizing Pancreatitis. Annals of Surgery.

[23] van Brunschot S, van Grinsven J, van Santvoort HC, Bakker OJ, Besselink MG, Boermeester MA, et al. Endoscopic or surgical step-up approach for infected necrotising pancreatitis: a multicentre randomised trial. Lancet 2018;391(10115):51-58. S0140-6736(17)32404-2 [pii]. 10.1016/S0140-6736(17)32404-2 29108721

[24] van Brunschot S, Fockens P, Bakker OJ, Besselink MG, Voermans RP, Poley JW (2014). Endoscopic transluminal necrosectomy in necrotising pancreatitis: a systematic review. Surg Endosc.

[25] van Brunschot S, Besselink MG, Boermeester MA, Gooszen HG, Horvath KD, van Santvoort HC (2013). Video-assisted retroperitoneal debridement (VARD) of infected necrotizing pancreatitis: an update. Curr Surg Rep.

[26] Hollemans RA, Bollen TL, van Brunschot S, Bakker OJ, Ahmed Ali U, van Goor H (2016). Predicting success of catheter drainage in infected necrotizing pancreatitis. Ann Surg.

