# Supplementary material for: Open necrosectomy in acute pancreatitis–obsolete or still useful?
Source: World J Emerg Surg. 2020 Mar 17;15:21. doi: 10.1186/s13017-020-00300-9 (PMC7079510; doi:10.1186/s13017-020-00300-9)
Supplement: Supplementary file 1 — Additional file 1:. Additional Table 1. Reoperations Within 6 Months and Endoscopic/Other Procedures Within One Year After First Necrosectomy. [file 13017_2020_300_MOESM1_ESM.docx]

**Additional Table 1.** Reoperations Within 6 Months and Endoscopic/Other Procedures Within One Year After First Necrosectomy

|  | **(n = 109)** |
| --- | --- |
| **Any reoperation** | 52 (47.7%) |
| Number of any reoperations, median (IQR, range) | 2 (1-3, 1-8) |
| **Renecrosectomy** | 27 (24.8%) |
| Number of renecrosectomies, median (IQR, range) | 1 (1-1, 1-4) |
| **Other reoperations** | 42 (38.5%) |
| Number of other reoperations, median (IQR, range) | 1 (0-2, 0-5) |
| **Indication for other operations** |  |
| Suspicion or verified enteric fistula/perforation | 16 (14.7%) |
| Pancreatic fistula | 9 (8.3%) |
| Bleeding, all | 16 (14.7%) |
| Bleeding within 1 week of first necrosectomy | 11 (10.1%) |
| Wound dehiscence | 7 (6.4%) |
| Biliary leakage/fistula | 3 (2.8%) |
| Infection | 2 (1.8%) |
| Suspicion or verified bowel ischaemia | 4 (3.7%) |
| Exploration and lavage (suspicion of bleeding) | 1 (0.9%) |
| Bowel continuity restoration | 1 (0.9%) |
| **Resections** |  |
| Resection of pancreas | 8 (7.3%) |
| Bowel resection | 14 (12.8%) |
| Cholecystectomy | 3 (2.8%) |
| Splenectomy | 5 (4.6%) |
| **Reconstructions** |  |
| Stoma formation | 10 (9.2%) |
| Operation of enteric fistula | 8 (7.3%) |
| Pancreatico-/fistulojejunostomy | 4 (3.7%) |
| Bile duct procedure * | 3 (2.8%) |
| **Time between first necrosectomy and removal of last percutaneous drainage tube, median (IQR, range), days** | 48 (26-85, 3-203) |
| **Postoperative radiologic drainage of collection** † | 28 (25.7%) |
| **Pancreatic fistula** ‡ | 43 (39.4%) |
| **Endoscopic or percutaneous procedures** |  |
| Any | 54 (49.5%) |
| ERP | 40 (36.7%) |
| ERC | 24 (22.0%) |
| Endoscopic stenting for pancreatic fistula | 32 (29.4%) |
| Endoscopic drainage of collection § | 8 (7.3%) |
| Endoscopic necrosectomy †† | 2 (1.8%) |
| Dilatation/stenting of duodenum | 3 (2.8%) |
| Endoscopic treatment for enteral fistula †† | 3 (2.8%) |
| Change of percutaneous drainage/fistulography | 25 (22.9%) |

* Hepaticojejunostomy or T-drainage of common bile duct.

† Percutaneous drainage performed by intervention radiologist within 6 months after first necrosectomy.

‡ Diagnosed within one year after first necrosectomy.

§ Pseudocystoventriculostomy (n=3), transpapillary drainage (n=4), nasocystic drainage tube (n=1).

†† Endoluminal or percutaneous.

IQR= Interquartile range, ERC= Endoscopic retrograde cholangiography, ERP= Endoscopic retrograde pancreatography
