# Supplementary material for: Open necrosectomy in acute pancreatitis–obsolete or still useful?
Source: World J Emerg Surg. 2020 Mar 17;15:21. doi: 10.1186/s13017-020-00300-9 (PMC7079510; doi:10.1186/s13017-020-00300-9)
Supplement: Supplementary file 2 — Additional file 2:. Additional Table 2. Univariate Analysis of 90-day Mortality. [file 13017_2020_300_MOESM2_ESM.docx]

**Additional Table 2.** Univariate Analysis of 90-day Mortality

| **Risk factor** | **Survivors (n = 84)** * | **Non-survivors (n = 25)** * | **OR (95% CI)** | ***P*** |
| --- | --- | --- | --- | --- |
| **Age, years** |  |  |  |  |
| <50 | 37 (44.0%) | 5 (20.0%) | Reference | **0.013** |
| 50-59 | 29 (34.5%) | 7 (28.0%) | 1.786 (0.514-6.212) | 0.362 |
| ≥60 | 18 (21.4%) | 13 (52.0%) | 5.344 (1.650-17.309) | **0.005** |
| **Male sex** | 75 (89.3%) | 21 (84.0%) | 0.630 (0.176-2.251) | 0.490 |
| **Co-morbidities** |  |  |  |  |
| Heart disease | 14 (16.7%) | 8 (32.0%) | 2.353 (0.851-6.509) | 0.153 |
| Pulmonary disease | 7 (8.3%) | 3 (12.0%) | 1.500 (0.358-6.287) | 0.693 |
| Mild renal insufficiency | 2 (2.4%) | 2 (8.0%) | 3.565 (0.476-26.709) | 0.225 |
| Diabetes | 8 (9.5%) | 3 (12.0%) | 1.295 (0.317-5.302) | 0.712 |
| Liver cirrhosis | None | 2 (8.0%) | Infinite (1.815-infinite) | 0.051 |
| Chronic pancreatitis | 2 (2.4%) | 1 (4.0%) | 1.708 (0.148-19.662) | 0.546 |
| None of the above | 55 (65.5%) | 11 (44.0%) | 0.414 (0.167-1.028) | 0.065 |
| **Necrosectomy <28 days from symptom onset** | 23 (27.4%) | 17 (68.0%) | 5.636 (2.142-14.831) | **<0.001** |
| **Open abdomen** † | 9 (10.7%) | 5 (20.0%) | 2.083 (0.628-6.912) | 0.304 |
| **Type of collection** |  |  |  |  |
| Walled off necrosis | 76 (90.5%) | 15 (60.0%) | 0.158 (0.054-0.466) | **0.001** |
| Acute necrotic collection | 8 (9.5%) | 10 (40.0%) | 6.333 (2.146-18.689) | **0.001** |
| Infected pancreatic necrosis | 69 (82.1%) | 16 (64.0%) | 0.386 (0.144-1.039) | 0.096 |
| **Revised Atlanta classification of severity** |  |  |  |  |
| Moderately severe | 32 (38.1%) | 7 (28.0%) | 0.632 (0.238-1.680) | 0.477 |
| Severe | 52 (61.9%) | 18 (72.0%) | 1.582 (0.595-4.207) | 0.477 |
| **Organ failure** ‡ |  |  |  |  |
| No organ failure | 56 (66.7%) | 8 (32.0%) | Reference | **<0.001** |
| Single organ failure | 11 (13.1%) | 1 (4.0%) | 0.636 (0.072-5.613) | 0.684 |
| Multiple organ failure | 17 (20.2%) | 16 (64.0%) | 6.588 (2.406-18.042) | **<0.001** |
| **Preoperative WBC count ≥23.0** § | 9 (10.7%) | 15 (60.0%) | 12.500 (4.342-35.989) | **<0.001** |
| **Step-up intervention** \|\| | 35 (41.7%) | 5 (20.0%) | 0.350 (0.120-1.022) | 0.060 |
| **Indication for necrosectomy** |  |  |  |  |
| Verified infected pancreatic necrosis ¶ | 49 (58.3%) | 8 (32.0%) | Reference | **<0.001** |
| Clinical suspicion of infected pancreatic necrosis | 20 (23.8%) | 5 (20.0%) | 1.531 (0.446-5.252) | 0.498 |
| Deterioration/prolonged organ failure | 6 (7.1%) | 12 (48.0%) | 12.250 (3.572-42.013) | **<0.001** |
| Other # | 9 (10.7%) | None | 0.000 (0.000-NC) | 0.999 |
| **Preoperative computed tomography** |  |  |  |  |
| Distant necrosis ** | 74 (88.1%) | 24 (96.0%) | 3.243 (0.395-26.659) | 0.451 |
| Disconnected left pancreatic remnant | 32 (38.1%) | 2 (8.0%) | 0.141 (0.031-0.640) | **0.003** |
| **Operative findings, first necrosectomy** |  |  |  |  |
| Infected pancreatic necrosis | 69 (82.1%) | 16 (64.0%) | 0.386 (0.144-1.039) | 0.096 |
| Peripancreatic necrosis only | 37 (44.0%) | 11 (44.0%) | 0.998 (0.406-2.454) | 1.000 |
| Pancreatic necrosis, no debridement | 2 (2.4%) | 5 (20.0%) | 10.250 (1.852-56.763) | **0.007** |
| Debridement of pancreatic necrosis | 45 (53.6%) | 9 (36.0%) | 0.488 (0.194-1.226) | 0.172 |
| Disconnected left pancreatic remnant | 11 (13.1%) | 2 (8.0%) | 0.577 (0.119-2.796) | 0.728 |
| Resection of pancreas | 10 (11.9%) | 2 (8.0%) | 0.643 (0.131-3.151) | 0.730 |
| **Operator acute care GI surgeon** | 75 (89.3%) | 19 (76.0%) | 0.380 (0.120-1.199) | 0.105 |

* Percentage in brackets is % of survivors/non-survivors.

† At the time of first necrosectomy.

‡ Cardiovascular, respiratory or renal, within 24 h of first necrosectomy.

§ WBC count expressed as 1 x 10^9^/L. One WBC count was not taken 24h prior to operation, and thus the last available WBC count prior to first necrosectomy was used.

|| Endoscopic, percutaneous or surgical drainage preceding index operation.

¶ Positive bacterial culture or gas on computed tomography scan.

# Prolonged symptoms (6), gastric outlet obstruction (1), bleeding (1) and colon necrosis suspicion (1).

** Local necrosis around pancreas, distant necrosis also in left/right paracolic gutter and/or retromesenteric area.

WBC=White blood cell, OR= Odds ratio, CI=Confidence interval, NC= Not countable.
